# Mass spectrometry protein expression profiles in colorectal cancer tissue associated with clinico-pathological features of disease

**DOI:** 10.1186/1471-2407-10-410

**Published:** 2010-08-06

**Authors:** Christopher CL Liao, Nicholas Ward, Simon Marsh, Tan Arulampalam, John D Norton

**Affiliations:** 1 Department of Biological Sciences, University of Essex, Wivenhoe Park, Colchester, Essex CO4 3SQ UK; 2 ICENI Centre, Department of Surgery, Colchester Hospital University NHS Foundation Trust, Turner Road, Colchester, Essex CO4 5JL UK

## Abstract

**Background:**

Studies of several tumour types have shown that expression profiling of cellular protein extracted from surgical tissue specimens by direct mass spectrometry analysis can accurately discriminate tumour from normal tissue and in some cases can sub-classify disease. We have evaluated the potential value of this approach to classify various clinico-pathological features in colorectal cancer by employing matrix-assisted laser desorption ionisation time of-flight-mass spectrometry (MALDI-TOF MS).

**Methods:**

Protein extracts from 31 tumour and 33 normal mucosa specimens were purified, subjected to MALDI-Tof MS and then analysed using the 'GenePattern' suite of computational tools (Broad Institute, MIT, USA). Comparative Gene Marker Selection with either a t-test or a signal-to-noise ratio (SNR) test statistic was used to identify and rank differentially expressed marker peaks. The *k*-nearest neighbours algorithm was used to build classification models either using separate training and test datasets or else by using an iterative, 'leave-one-out' cross-validation method.

**Results:**

73 protein peaks in the mass range 1800-16000Da were differentially expressed in tumour *verses *adjacent normal mucosa tissue (P ≤ 0.01, false discovery rate ≤ 0.05). Unsupervised hierarchical cluster analysis classified most tumour and normal mucosa into distinct cluster groups. Supervised prediction correctly classified the tumour/normal mucosa status of specimens in an independent test spectra dataset with 100% sensitivity and specificity (95% confidence interval: 67.9-99.2%). Supervised prediction using 'leave-one-out' cross validation algorithms for tumour spectra correctly classified 10/13 poorly differentiated and 16/18 well/moderately differentiated tumours (*P *= < 0.001; receiver-operator characteristics - ROC - error, 0.171); disease recurrence was correctly predicted in 5/6 cases and disease-free survival (median follow-up time, 25 months) was correctly predicted in 22/23 cases (*P *= < 0.001; ROC error, 0.105). A similar analysis of normal mucosa spectra correctly predicted 11/14 patients with, and 15/19 patients without lymph node involvement (*P *= 0.001; ROC error, 0.212).

**Conclusions:**

Protein expression profiling of surgically resected CRC tissue extracts by MALDI-TOF MS has potential value in studies aimed at improved molecular classification of this disease. Further studies, with longer follow-up times and larger patient cohorts, that would permit independent validation of supervised classification models, would be required to confirm the predictive value of tumour spectra for disease recurrence/patient survival.

## Background

Colorectal cancer (CRC) is the second commonest malignancy and has a five-year survival rate of approximately 50% [1,2]. The majority of patients, particularly with early stage disease (Dukes' A, Stage I), are treated with surgery [3]. For more advanced disease (Dukes' C and D, Stage III or IV) surgery combined with adjuvant chemotherapy has proven survival benefits [4-6]. However, the disease outcome is very variable and prognosis and prediction of treatment response based on conventional disease staging criteria is not reliable [6,7]. There has therefore been considerable interest in the development of more robust prognostic and predictive disease markers for patient stratification with the ultimate aim of tailoring treatment to the individual patient [8,9].

Markers based on circulating carcinoembryonic antigen (CEA) levels and various tumour-associated gene mutations including microsatellite instability (MSI), loss of heterozygosity of 18q, deleted in colorectal cancer (DCC), mutations in *KRAS*, *BRAF *and *PIK3CA *genes have all been shown to be of some prognostic or predictive value (reviewed in [8,10]). In particular, the mutational status of *KRAS*, *BRAF *and *PIK3CA *genes has recently been proposed as a reliable marker for predicting responders to new targeted agents for the epidermal growth factor receptor (EGFR) [11,12]. In addition, gene expression profiling studies of both mRNA [13] and microRNA [14] have revealed tumour-associated gene expression signatures that form the basis for a molecular classification of disease sub-types that define disease course and treatment response (reviewed in [8]). These studies on gene mutations and RNA expression have been paralleled by analysis of the tumour cell proteome, most commonly employing the technique of two-dimensional difference gel electrophoresis (2D-DIGE) to identify proteins that are differentially expressed in tumour *verses *normal mucosa tissue (reviewed in [15]). An expanding list of candidate prognostic markers have emerged from these studies including for example, cathepsin D, S100A4 and APAF-1 [15].

As an alternative to 2D-DIGE, studies of other tumour types have also employed the technique of direct protein expression profiling of tumour/normal tissue by surface enhanced laser desorption ionisation time-of-flight mass spectrometry (SELDI-TOF) or by matrix-assisted laser desorption ionisation time of-flight-mass spectrometry (MALDI-TOF) mass spectrometry [16,17]. This approach, which is most commonly associated with the development of serum-based diagnostic markers, offers a number of advantages over 2D-DIGE. Although the technique yields no information on the actual identities of proteins, the reproducible spectral profiles that are relatively simple to generate in high throughput studies allow robust classification models of different proteome populations to be built. For example, studies of lung [18], breast [19], head and neck cancer [20] have all shown that the spectral profiles of tumour and normal tissue can be accurately discriminated and in some cases sub-classified by direct protein profiling using SELDI/MALDI-TOF mass spectrometry. Only one previous study has reported on the detection of differences between normal mucosa, adenoma and colorectal carcinoma by using SELDI-TOF MS [21].

In the present study, we have evaluated the potential value of protein expression profiling of CRC tissue by MALDI-TOF mass spectrometry. In addition to comparing tumour with adjacent normal mucosa, we have investigated whether spectral profiles of tumour tissue can be used to classify various clinico-pathological features of disease. Since previous 2D-DIGE studies have reported abnormalities of protein expression profiles in tumour-adjacent normal tissue [22], we have also extended this analysis to normal mucosa tissue.

## Methods

### Clinical specimens

Tissue samples were collected from a total of 36 patients with confirmed CRC at the time of surgical resection at Colchester General Hospital, Essex UK. All specimens were obtained following informed consent in accordance with local UK NHS Ethics Committee approval (protocol reference: MH 528). Surgically excised specimens were washed extensively in ice-cold 150 mM NaCl and samples of normal colonic mucosa (>10 cm from tumour margin) and tumour tissues were excised using a scalpel and then snap frozen and transferred to a - 80°C freezer. The total time from surgical resection to snap freezing of specimens was <30 mins.

### Protein extraction and purification

Frozen tissue samples (approximately 250 mg) were ground using a mortar and pestle and then lysed for 30 mins at 4°C in 1.0 ml of 10 mM Tris-HCl pH 7.5, 200 mM NaCl containing Protease inhibitor cocktail (Roche Pharmaceuticals) and 1% N-octyl-β-D-glucopyranoside (Sigma Aldrich). The cell lysate was then centrifuged at 12,000 × g for 30 mins and the supernatant representing the solubilised fraction was removed. Protein was further purified by reversed phase hydrophobic interaction chromatography using a commercially available super-paramagnetic microparticle kit (MB-HIC-C8, Bruker Daltonics). Briefly, 10 μl of 30-35 mg/ml protein solution was adsorbed to 10 μl of beads after addition of 20 μl kit binding buffer. After three washes with 200 μl 0.1% trifluoroacetic acid, protein was eluted in 20 μl of 50% (v/v) acetonitrile (Fisher Scientific) Eluted protein was stored at 4°C for no more than 1 hr prior to matrix co-crystallisation.

### MALDI-TOF mass spectrometry

To facilitate reproducible co-crystallisation of protein with matrix solution, a modification of the slow crystallisation method [23] was used. Briefly, 20 ul of purified protein was mixed with 20 μl of acetonitrile containing 0.1% trifluoroacetic acid, saturated with sinapic acid (Sigma Aldrich). A 20 μl aqueous solution containing diammonium citrate (200 mM) and nitrotetracetic acid (0.1%) was added and crystal formation was allowed to proceed for 2-3 hrs. Crystallised matrix-protein samples were spotted onto a stainless steel MALDI target plate and spectra were acquired using a MALDI-TOF mass spectrometer (Reflex IV; Bruker Daltonics) with the following instrument settings: ion source 1, 20 kV; ion source 2, 16.65 kV; lens voltage, 9.5 kV; pulsed ion extraction, 200 ns. Ionisation was achieved by irradiation with a nitrogen laser (e = 337 nm) operating at 25 Hz and 20% laser power. For matrix suppression, we used a high gating factor with signal suppression up to 1500 Da. Mass spectra were detected in linear positive mode. Detector gain was set at 1600 V, sample rate at 1.0 and electronic gain at 100 mV with real-time smoothing. Spectra were acquired in duplicate from 500 laser shots delivered as 5 × 100 pulses and were internally calibrated using 'FlexAnalysis' spectral processing software (Version 2.0; Bruker Daltonics) with reference marker peaks at 2426.9Da, 6109.5 Da and 12471.6 Da. External calibration used the following reference standards: bombesin (1620.86 Da), somatostatin (3149.57 Da), insulin (5734.51 Da), ubiquitin I (8565.76 Da), cytochrome c (12,360.97 Da) and myoglobin (16,952.30 Da).

### Spectral processing and analysis

Calibrated spectra were exported as ASCII files and were digitally processed by smoothing, de-noising, baseline subtraction and normalisation (by total ion current) using the 'SpecAlign' suite of spectral computational tools [24,25]. Validation of the reproducibility of the resulting mass spectrometry profiles and elimination of 'outliers' was accomplished as described elsewhere [26]. Duplicate spectra with a cross-correlation function of < 0.950 were discarded. From the initial cohort of specimens, representing matched tumour and adjacent normal mucosa from 36 patients, a total of 64 spectra representing 31 tumours and 33 normal mucosa were obtained (see Table 1). Of the 5 tumour and 3 mucosa specimens that were excluded from analysis, 2 tumour and one mucosa failed to yield reproducible spectra on repeated protein preparations. The remaining 3 tumour and 2 mucosa specimens consistently gave spectra of poor quality (outliers), presumably as a result of specimen deterioration. Matching peaks were aligned across spectra by using the combined Fast Fourier Transform/Peak matching method [25] and modelled peak areas for the entire set of spectra were exported as a single csv file.

**Table 1:** Clinico-pathological features of patient specimens

Tumour	^1^NM	Age	Gender	Dukes' stage	TNM stage	Differentiation	Vascular invasion	^2^LNs harvested	LNs pos	Patient status	^3^Follow-up time
-	001NM	78	F	B	pT3, pN0, pR0	Poor	Absent	15	0	Well & symptom free	48

002T	002NM	91	M	B	pT3, pN0, pR0	Moderate	Absent	9	0	Deceased (recurrence)	35

003T	003NM	75	M	C1	pT3, pN1, pR0	Poor	Absent	10	3	Well & symptom free)	36

004T	004NM	74	F	C1	pT4, pN1, pR2	Poor	Present	11	3	Deceased (recurrence)	<1

005T	005NM	76	M	B	pT3, pN0, pR0	Poor	Absent	6	0	Well & symptom free	49

-	006NM	69	F	A	pT2, pN0, pR0	Well	Absent	11	0	Well & symptom free	40

-	007NM	52	M	C1	pT3, pN1, pR0	Poor	Absent	23	3	Well & symptom free	48

008T	008NM	63	F	C1	pT4, pN0, pR0	Poor	Absent	10	0	Deceased (recurrence)	40

009T	009NM	68	M	B	pT3, pN0, pR0	Poor	Absent	8	8	Well & symptom free	36

011T	011NM	77	M	C1	pT4,p N1, pR0	Poor	Absent	15	3	Well & symptom free	40

016T	016NM	61	M	C2	pT2, pN2, pR0	Moderate	Present	14	5	Well & symptom free	43

017T	017NM	65	F	B	pT3, pN0, pR0	Moderate	Absent	14	0	Well & symptom free	39

020T	020NM	65	F	B	pT3, pN0, pR0	Poor	Absent	12	0	Well & symptom free	36

021T	021NM	72	M	B	pT4, pN1, pR0	Moderate	Present	5	1	Well & symptom free	28

023T	023NM	59	M	B	pT3, pN0, pR0	Moderate	Absent	10	0	Well & symptom free	20

024T	024NM	41	F	C2	pT4, pN1, pRx	Well	Absent	15	2	Deceased (recurrence)	30

025T	-	82	M	B	pT4, pN0, pMx, pRx	Poor	Absent	7	0	Deceased (recurrence)	13

026T	026NM	76	F	A	pT2, pN0, pR0	Moderate	Absent	5	0	Deceased (recurrence)	36

028T	028NM	86	F	C1	pT3, pN1, pR0	Moderate	Absent	12	0	Well & symptom free	36

029T	029NM	71	F	B	pT3, pN0, pR0	Well	Absent	32	0	Well & symptom free	36

031T	031NM	82	M	C2	pT3, pN2, pR0	Poor	Present	11	3	Well & symptom free	36

032T	032NM	69	F	B	pT4, pN0, pR0	Moderate	Absent	11	0	Well & symptom free	23

033T	033NM	72	M	C1	pT4, pN1, pR0	Moderate	Absent	8	1	Well & symptom free	22

034T	034NM	58	M	C1	pT4, pN1, pR0	Moderate	Absent	5	3	Well & symptom free	25

-	035NM	77	F	B	pT3, pN0, pR0	Poor	Absent	7	0	Well & symptom free	25

036T	-	81	F	B	pT3, pN0, pR0	Moderate	Absent	13	0	Well & symptom free	21

037T	037NM	77	F	B	pT3, pN0, pR0	Well	Absent	7	0	Well & symptom free	19

038T	038NM	76	F	A	pT2, pN1, pR0	Poor	Absent	5	1	Well & symptom free	20

039T	039NM	75	F	B	pT3, pN0, pR0	Moderate	Absent	16	0	Well & symptom free	23

2012T	2012NM	62	M	C1	pT3, pN1, pR0	Poor	Present	18	3	Well & symptom free	20

2018T	2018NM	83	F	A	pT1, pN0, pR0	Moderate	Absent	6	0	Deceased (unrelated)	2

2022T	2022NM	56	M	B	pT3, pN0, pR0	Well	Present	20	0	Well & symptom free	20

2044T	2044NM	82	M	A	pT2, pN0, pR0	Moderate	Absent	10	0	Well & symptom free	21

-	2080NM	72	F	A	pT2, pN0, pR0	Moderate	Absent	5	0	Well & symptom free	21

2084T	-	38	M	B	ypT3, ypN0, ypR0	Poor	Absent	10	0	Well & symptom free	20

2085T	2085NM	78	F	C1	pT3, pN1, pR0	Moderate	Absent	11	1	Deceased (unrelated)	<1

^1^NM = normal mucosa; ^2^LN = lymph node; ^3^follow-up time in months

Subsequent spectral analysis was implemented in the 'GenePattern' suite of software tools (Broad Institute, MIT, USA) [27]. Hierarchical clustering used Euclidean correlation as the column distance measure with pair-wise average linkage as the clustering method. Comparative Gene Marker Selection [28,29] with either a t-test or a signal-to-noise ratio (SNR) test statistic was used to identify and rank differentially expressed marker peaks and to assign Bonferroni-corrected *P *and false discovery rate (FDR) values [28-30]. The *k*-nearest neighbours (*k*NN) algorithm [29] was used to build a classification model for tumour *vs *normal using separate training and test datasets. For this purpose, two thirds of the spectra, comprised of a representative proportion of tumour and normal spectra, were randomly assigned to a training dataset, with the remaining third being used as an independent test dataset. Spectra were randomly assigned using the GenePattern 'SplitDatasetTrainTest' module [27]. Alternatively the *k*NN algorithm was used in an iterative, 'leave-one-out' cross-validation mode. Other statistical analysis used the SPSS software.

## Results

### Spectral profiles in tumour and normal mucosa tissues

**Figure 1:**
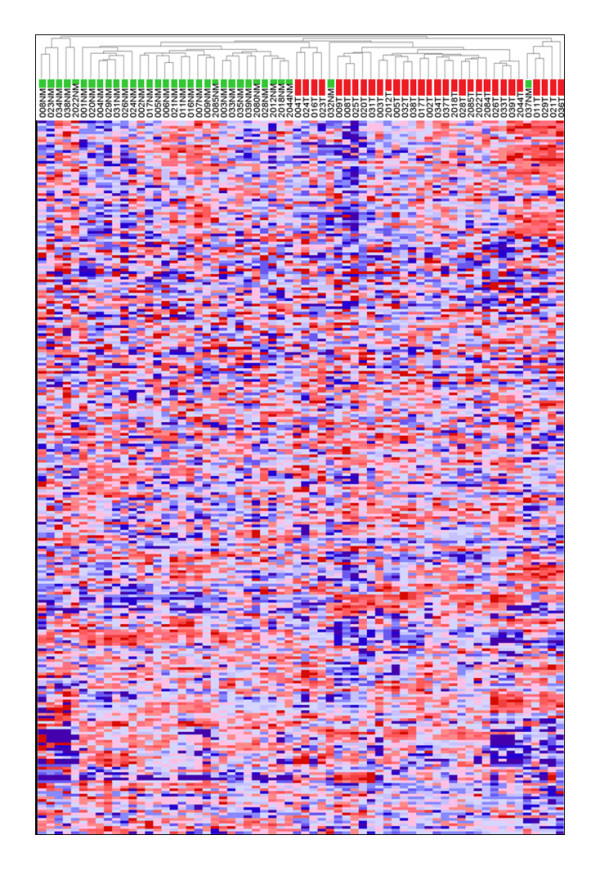
**Unsupervised hierarchical cluster analysis of tumour and normal mucosa spectra**. The dendrogram and heatmap show the clustering of Tumour (T) and normal mucosa (NM) spectra using Euclidean correlation as the column distance measure with pair-wise average linkage as the clustering method. Row clustering (not shown) used Spearman's rank correlation as distance measure with pair-wise complete linkage as the clustering method. Specimens are colour-coded as green (NM) and red (T).

Table 1 summarises the clinico-pathological data for the 36 CRC patients from whom specimens were obtained. In most cases, spectra of adequate quality from matching pairs of tumour and adjacent normal mucosa were obtained. However, some tissue protein preparations consistently yielded spectra of poor quality or that were poorly reproducible (see Methods section); these were excluded from the analysis. The resulting 64 spectra, representing 31 tumour and 33 normal mucosa specimens, generated a total of 265 protein peaks in the mass range 1800-16000Da. Illustrative examples of raw MALDI-TOF spectral profiles are shown in additional file 1. Although the overall intensity profile of individual protein peaks was very heterogeneous across different specimens, unsupervised hierarchical cluster analysis classified most tumour and normal mucosa into distinct cluster groups (Figure 1) consistent with major differences in the tumour *verses *normal protein expression profiles.

**Figure 2:**
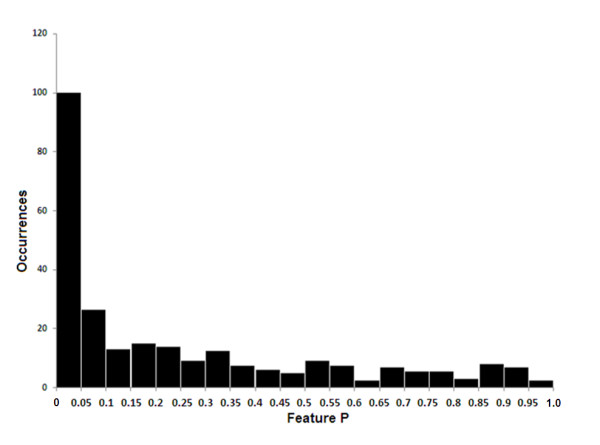
**Probability distribution of marker peaks distinguishing tumour from normal mucosa**. Spectra from all 64 tumour and normal tissue samples were analysed by Comparative Gene Marker Selection [28] using the SNR test statistic to identify peaks (features) that discriminate tumour from normal tissue. The feature P histogram shows the number of peaks (occurrences) that fall within binned *P *values.

**Figure 3:**
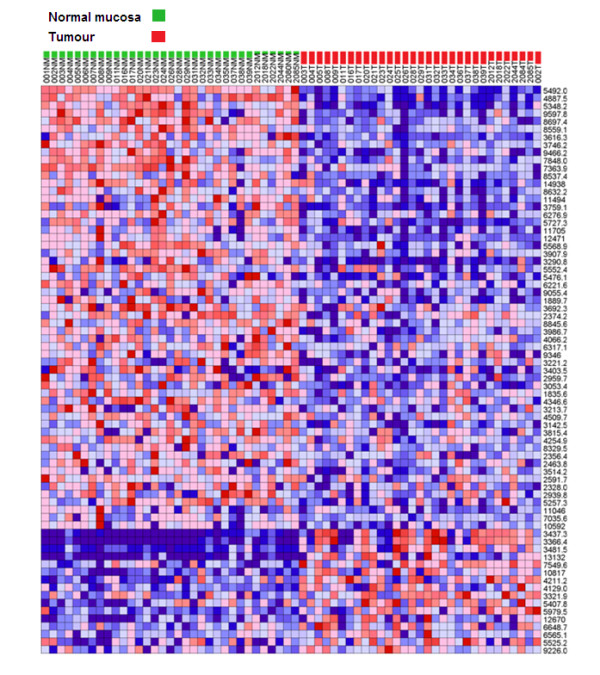
**Heat map profile of marker peaks discriminating tumour from normal mucosa**. The expression profiles and m/z values of the top 73 ranked peaks identified by Comparative Gene Marker Selection [28] (*P *= ≤ 0.01, FDR = ≤ 0.05) are depicted for all 64 tissue specimens.

To quantitatively evaluate the differences between the protein expression profiles of tumour *verses *normal tissue, the Comparative Gene Marker Selection algorithm [28] was applied to the spectral data-set to determine the level of significance of difference between tumour and normal for each protein peak. Figure 2 shows the frequency distribution (occurrences) of protein peak *P *values (Feature P) that were binned in increments of 0.05. Above *P *= 0.05, the representation of protein peaks was fairly evenly distributed. However, nearly 100 peaks gave a *P *value < 0.05, indicating that a sizable fraction of proteins detected by MALDI-TOF mass spectrometry discriminate between tumour and normal colonic tissue. Applying a threshold of *P *≤ 0.01, FDR ≤ 0.05, the expression profile of a total of 73 protein peaks was significantly different between tumour and normal tissue with 57 being up-regulated in normal tissue and 16 being up-regulated in tumour tissue. Figure 3 shows a heat-map profile of these 'marker peaks' and additional file 2 summarises their statistical features.

To rigorously demonstrate that tumour and normal mucosa tissue could be distinguished using their protein spectral profiles, the 64 spectra were randomly split into separate training and test datasets. The training spectra dataset was used to optimise a *k*NN algorithm [29] for predicting tumour or normal status. As summarised in additional file 3, the model correctly predicted the status of specimens in the independent test spectra dataset with 100% sensitivity and specificity (95% confidence interval: 67.9-99.2%).

### Classification of clinico-pathological characteristics from tumour spectra

**Figure 4:**
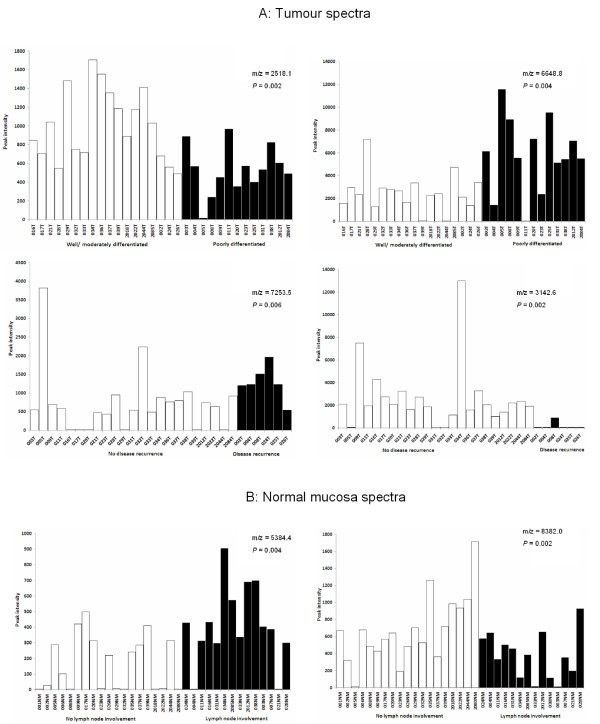
**Relative ion intensity profiles of marker peaks used in predictive algorithms of tumour/mucosa clinico-pathological features**. The peak intensity profiles of the top two-ranked scoring peaks are shown for tumour spectra (A) for classifying differentiation and disease recurrence and for normal mucosa spectra (B) for classifying lymph node involvement (see Table 2). The performance of predictive models for these clinico-pathological features are shown in additional file 4 (differentiation), in additional file 5 (disease recurrence) and in additional file 6 (lymph node involvement). The t-test *P *value is given for each marker peak.

To determine whether the protein expression profiles of tumour tissue could be used to predict individual clinico-pathological characteristics of patients (Table 1), the *k*NN algorithm was used to optimise a series of classification models. Since the limited numbers of datasets precluded analysis by using independent train and test spectra, the *k*NN algorithm was used in an iterative, 'leave-one-out' cross-validation mode. Table 2 summarises the results of this analysis. The predictive model for distinguishing poorly differentiated from well/moderately differentiated tumours gave a receiver-operator characteristics (ROC) error of 0.171, correctly classifying 10/13 poorly differentiated and 16/18 well/moderately differentiated tumours (*P *= < 0.001). Additional file 4 summarises the *k*NN algorithm results and Figure 4A shows the expression profiles of the top two ranked discriminating peaks. The *k*NN model for disease recurrence also gave a low ROC error (0.105 - see Table 2A). As summarised in additional file 5, the model correctly predicted 5/6 patients with recurrent disease and 22/23 who are disease-free (*P *= < 0.001). Figure 4A shows the expression profiles of the top two ranked marker peaks for classifying disease outcome.

**Table 2:** Performance of predictive models for classification of clinico-pathological characteristics in tumour tissue

CHARACTERISTICS	^1^Advanced Dukes' stage	Poorly differentiated	Lymph node involvement	Invasiveness	^2^Disease recurrence
Number of features	5	2	4	9	10

Positive prediction rate	6/12	10/13	5/13	3/7	5/6

Sensitivity	0.500	0.769	0.385	0.429	0.833

^3^CI	0.223-0.777	0.460-0.938	0.151-0.677	0.118-0.798	0.364-0.991

Positive predictive value	0.750	0.833	0.625	0.750	0.833

CI	0.356-0.955	0.509-0.971	0.259-0.898	0.219-0.986	0.364-0.991

Negative prediction rate	17/19	16/18	15/18	23/24	22/23

Specificity	0.894	0.889	0.833	0.958	0.957

CI	0.654-0.981	0.639-0.981	0.577-0.956	0.768-0.998	0.760-0.998

Negative predictive value	0.739	0.842	0.652	0.852	0.957

CI	0.513-0.889	0.585-0.958	0.428-0.828	0.654-0.951	0.760-0.998

Absolute error	0.258	0.161	0.355	0.161	0.069

^4^ROC error	0.302	0.171	0.391	0.307	0.105

Fisher's exact test	*P *= 0.020	*P *= < 0.001	*P *= 0.133	*P *= 0.027	*P *= < 0.001

^1^Includes Dukes' C1 and C2; ^2 ^Median follow-up time for recurrent disease patients: 33 months; median follow-up time for disease-free patient: 27 months (analysis excludes patients who died through surgical complications - see Table 1); ^3^CI = 95% confidence interval; ^4^ROC = receiver-operator characteristics

The KNN algorithm [29] was used in 'leave-one-out' cross-validation prediction with the number of features (marker peaks) specified. Marker peaks were selected using a t-test statistic except for lymph node involvement and invasiveness characteristics of tumour tissue where the SNR test statistic was used.

### Classification of clinico-pathological characteristics from normal mucosa spectra

In a similar analysis of normal mucosa spectra (Table 3), only the characteristic of lymph node involvement gave a low ROC error (0.212). As shown in Table 3 and in additional file 6, the *k*NN algorithm correctly predicted 11/14 patients with, and 15/19 patients without lymph node involvement (*P *= 0.001). Figure 4B shows the expression profiles of the top two ranked marker peaks for classifying the characteristic of lymph node involvement.

**Table 3:** **Performance of predictive models for classification of clinico-pathological characteristics in normal mucosa tissue**.

CHARACTERISTICS	^1^Advanced Dukes' stage	Poorly differentiated	Lymph node involvement	Invasiveness	^2^Disease recurrence
Number of features	7	5	3	6	7

Positive prediction rate	8/13	8/14	11/14	3/7	0/5

Sensitivity	0.615	0.571	0.786	0.429	0.000

^3^CI	0.322-0.849	0.296-0.812	0.488-0.943	0.116-0.798	0.000-0.537

Positive predictive value	0.500	0.444	0.733	0.500	0.000

CI	0.255-0.749	0.224-0.686	0.448-0.911	0.139-0.860	0.000-0.945

Negative prediction rate	12/20	9/19	15/19	23/26	25/26

Specificity	0.600	0.474	0.789	0.885	0.962

CI	0.364-0.800	0.252-0.705	0.539-0.930	0.687-0.970	0.784-0.998

Negative predictive value	0.706	0.600	0.833	0.852	0.833

CI	0.440-0.886	0.329-0.825	0.577-0.956	0.654-0.951	0.645-0.937

Absolute error	0.394	0.485	0.212	0.212	0.194

^4^ROC error	0.392	0.477	0.212	0.343	0.519

Fisher's exact test	*P *= 0.139	*P *= 0.267	*P *= 0.001	*P *= 0.082	*P *= 0.839

^1^Includes Dukes' C1 and C2; ^2 ^Median follow-up time for recurrent disease patients: 33 months; median follow-up time for disease-free patient: 27 months (analysis excludes patients who died through surgical complications - see Table 1); ^3^CI = 95% confidence interval; ^4^ROC = receiver-operator characteristics

The KNN algorithm [29] was used in 'leave-one-out' cross-validation prediction with the number of features (marker peaks) specified. Marker peaks were selected using a t-test statistic except for lymph node involvement and invasiveness characteristics of tumour tissue where the SNR test statistic was used.

## Discussion

Although previous studies employing 2D-DIGE analysis of CRC tissues have documented a number of proteins that are either up- or down-regulated in tumour *verses *normal mucosa [15], the extent to which protein expression profile differences can be detected by direct MALDI-TOF analysis in CRC was not previously known. Analysis of complex protein mixtures by MALDI-TOF MS is inherently limited by the resolution afforded by this type of instrument. Also, only a minor fraction of protein species are efficiently ionisable and therefore detectable. However, our results show that, in common with similar studies in some other solid tumour types [18-20], MALDI-TOF MS readily detects a sizable fraction of protein marker peaks whose expression level is significantly different between tumour and normal mucosa. By using an optimised *k*NN training model, the classification of tumour and normal tissue was correctly predicted with 100% sensitivity and specificity (95% confidence interval: 0.679-0.992) in an independent test dataset. This performance compares favourably with other studies, for example in head and neck squamous cell carcinoma, in which supervised prediction using SELDI-TOF spectral data correctly classified healthy mucosa and tumour tissue with an accuracy of 94.5% and 92.9% respectively [20].

In further evaluating the potential value of spectra generated from tumour tissue for classifying various clinic-pathological characteristics of disease, we observed low ROC errors with the *k*NN predictive models for differentiation (0.171) and disease recurrence (0.105). Since histological differentiation stage is a characteristic that is intrinsic to the tumour tissue (and would most closely reflect the actual tumour cell proteome), the ability of the spectra to discriminate well/moderately differentiated from poorly differentiated histologies is perhaps unsurprising. The good performance of the predictive model for disease recurrence is consistent with data from several microarray expression profiling studies that have clearly demonstrated associations between patterns of tumour-associated gene expression and prognosis/treatment response [8,13,14]. However, given that in our study, only six patients had succumbed to recurrent disease at the time of data analysis (median follow-up time for recurrent disease patients: 33 months; median follow-up time for disease-free patient: 27 months), our results should be interpreted with caution. It is also important to emphasise that because of the relatively small number of tumour specimens, rigorous validation of correlations with disease recurrence and histological differentiation stage in an independent 'test' datsaset was not possible in our study.

Several lines of evidence indicate that the normal mucosa from surgically resected CRC tumour specimens display abnormalities in gene and protein expression. These abnormalities have been attributed to precancerous 'field effect' changes in tumour-adjacent mucosa and have been reported to affect protein expression [22], CpG island gene methylation [31] and gene microarray expression profiles [32]. Indeed one study has reported that gene expression profiling of non-neoplastic mucosa may predict clinical outcome of CRC patients [32]. These findings are reminiscent of reports from studies of other solid tumour types, most strikingly in hepatocellular carcimoma in which gene expression patterns of non-neoplastic liver tissue were predictive of patient survival, whereas tumour tissue gene expression signatures were of no prognostic value [33]. It was therefore of interest in our study to determine whether the protein expression profiles of normal mucosa could be used to classify any clinico-patholgical characteristics. Although we found no evidence for predictive value for disease relapse (ROC error, 0.519), the *k*NN model of normal mucosa spectra for lymph node involvement did give a low ROC error (0.212); the corresponding *k*NN model for tumour spectra did not show predictive value (0.391). One plausible scenario to explain the predictive value of normal mucosa spectra for lymph node involvement is that paracrine/inflammatory mechanisms, involving proximal affected lymph nodes, may induce changes to the microenvironment of tumour-adjacent mucosa.

As an essential pre-requisite for marker validation, it would be highly desirable in future studies to determine the identities of candidate marker peaks in tumour tissue that discriminate different histological differentiation stages and predict disease recurrence. Our findings also indicate that similar studies using the alternative approach of liquid chromatography coupled to tandem mass spectrometry (LC-MS/MS) in CRC are warranted.

## Conclusions

In summary, our study has shown that direct protein expression profiling of surgically resected CRC tissue by MALDI-TOF mass spectrometry has potential value in studies aimed at improved molecular classification of this disease. Further studies, with longer follow-up times and larger patient cohorts, that would permit independent validation of predictive models, would be required to confirm the predictive value of tumour spectra for disease recurrence/patient survival.

## Competing interests

The authors declare that they have no competing interests.

## Authors' contributions

CCLL collected specimens, processed all samples and collated and analysed data. NW collected specimens and collated data. SM and TA contributed to the study design and in arrangements for specimen collection. JDN contributed to the study design, mass spectrometry and data analysis and wrote the manuscript. All authors have read and approved the final manuscript.

## Pre-publication history

The pre-publication history for this paper can be accessed here:

http://www.biomedcentral.com/1471-2407/10/410/prepub

## Supplementary Material

Additional file 1 **Examples of raw MALDI-TOF spectral profiles**. Illustrative examples shown for 2012NM and 020T Click here for file

Additional file 2 **Summary of marker peaks discriminating tumour from normal mucosa**. Compilation of m/z values, ranking and statistics for 73 marker peaks. Click here for file

Additional file 3 **Performance of predictive model for discriminating tumour and normal mucosa**. Summary of results of optimised *k*-NN algorithm on an independent test dataset. Click here for file

Additional file 4 **Performance of model for predicting poor differentiation based on tumour spectra**. Summary of results of 'leave-one-out' cross-validation *k*-NN algorithm. Click here for file

Additional file 5 **Performance of model for predicting disease recurrence based on tumour spectra**. Summary of results of 'leave-one-out' cross-validation *k*-NN algorithm. Click here for file

Additional file 6 **Performance of model for predicting lymph node involvement based on mucosa spectra**. Summary of results of 'leave-one-out' cross-validation *k*-NN algorithm. Click here for file
