# Supplementary material for: Mass spectrometry protein expression profiles in colorectal cancer tissue associated with clinico-pathological features of disease
Source: BMC Cancer. 2010 Aug 6;10:410. doi: 10.1186/1471-2407-10-410 (PMC2927547; doi:10.1186/1471-2407-10-410)

Additional file 1: Examples of raw MALDI-TOF spectral profiles. The m/z scale is shown on the x axis; ion current is shown on the y axis.

Normal Mucosa (2012NM)

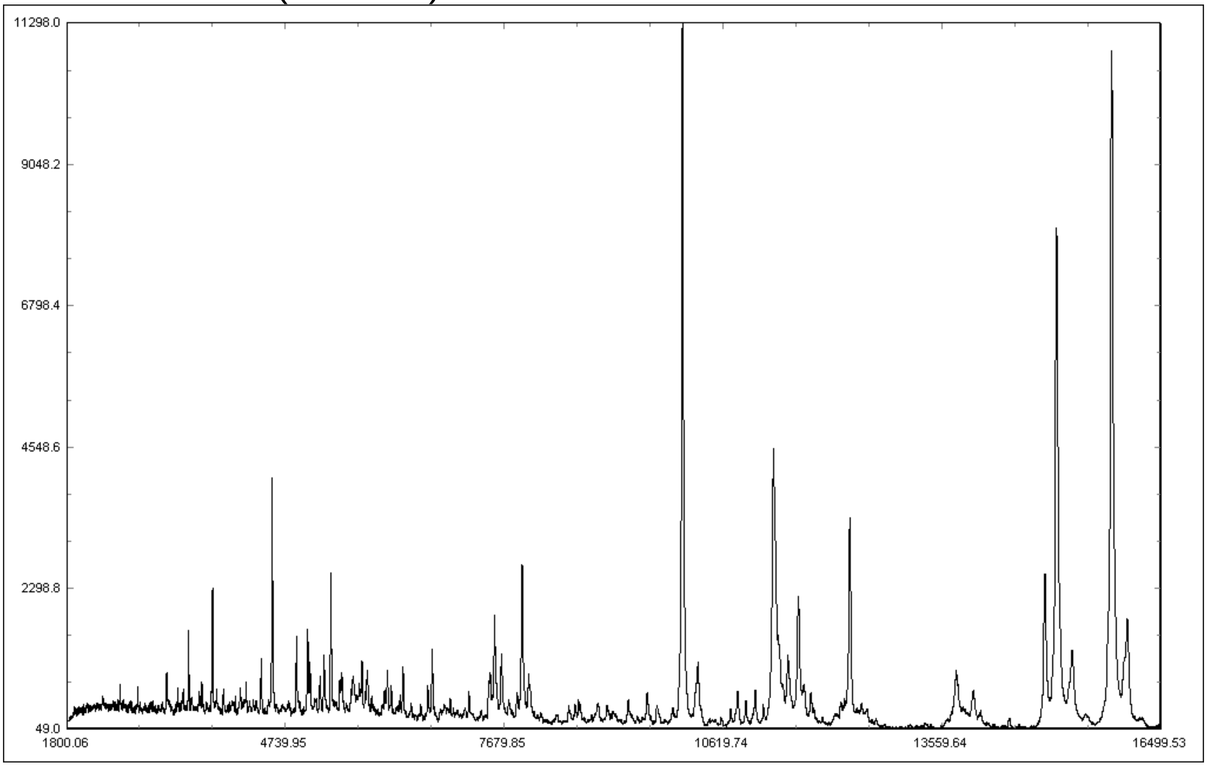

Tumour (020T)

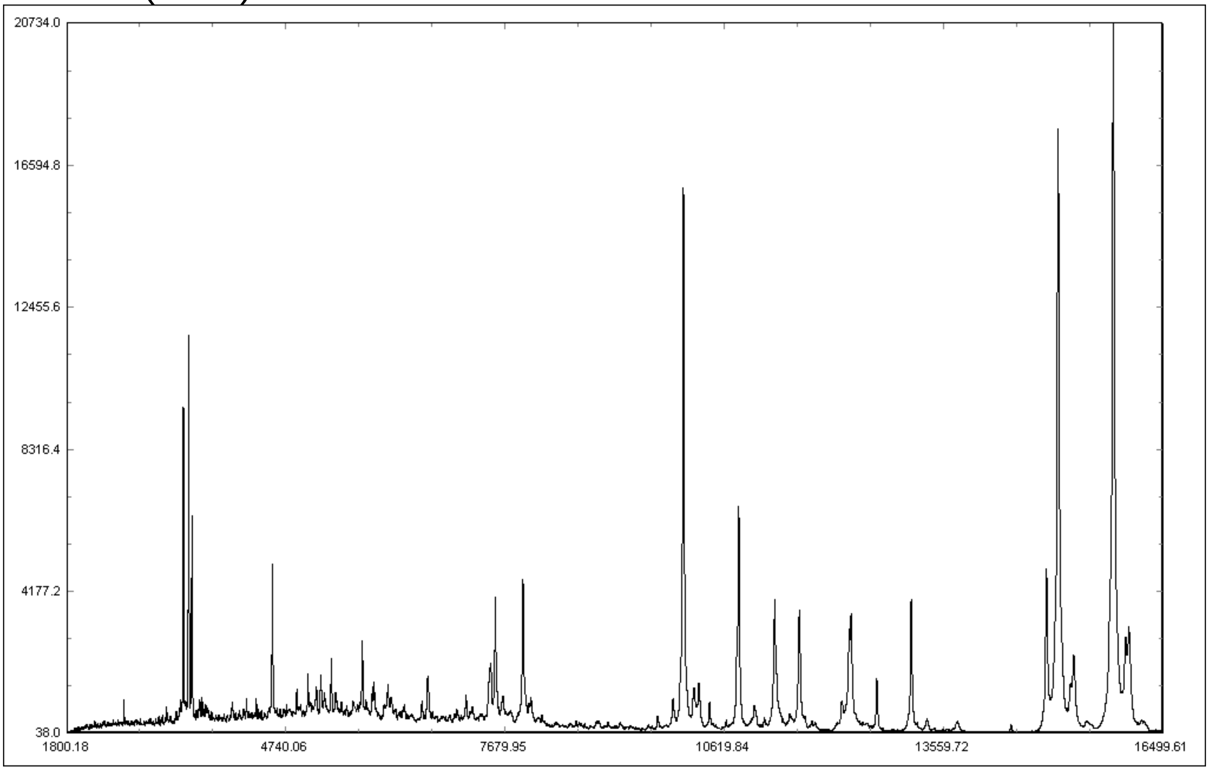

Supplement: Additional file 1 — Examples of raw MALDI-TOF spectral profiles. Illustrative examples shown for 2012NM and 020T [file 1471-2407-10-410-S1.PDF]
