# Supplementary material for: Mass spectrometry protein expression profiles in colorectal cancer tissue associated with clinico-pathological features of disease
Source: BMC Cancer. 2010 Aug 6;10:410. doi: 10.1186/1471-2407-10-410 (PMC2927547; doi:10.1186/1471-2407-10-410)
Supplement: Additional file 2 — Summary of marker peaks discriminating tumour from normal mucosa. Compilation of m/z values, ranking and statistics for 73 marker peaks. [file 1471-2407-10-410-S2.PDF]

Additional file 2: Summary of marker peaks discriminating tumour from normal mucosa. The list of marker peaks (features) and their corresponding m/z values that best distinguish tumour from normal mucosa were extracted by Comparative Gene Marker Selection [28] using the SNR test statistic. The rank order, based on values of the test statistic (score) is shown for peaks up-regulated in normal (N) or tumour (T) tissue.

rank	upreg. in	m/z value	score	[1]feature *P*	[2]FDR	[3]Fold change
1	N	5492.1	1.486	< 0.002	< 0.013	3.296
4	N	4887.5	1.059	< 0.002	< 0.013	3.139
5	N	5348.3	0.892	< 0.002	< 0.013	3.293
6	N	9597.9	0.874	< 0.002	< 0.013	3.058
7	N	8697.4	0.853	< 0.002	< 0.013	2.473
8	N	8559.1	0.723	< 0.002	< 0.013	1.747
10	N	3616.3	0.719	< 0.002	< 0.013	3.132
11	N	3746.2	0.705	< 0.002	< 0.013	1.762
12	N	9466.3	0.701	< 0.002	< 0.013	2.884
13	N	7848.0	0.682	< 0.002	< 0.013	2.341
14	N	7364.0	0.676	< 0.002	< 0.013	1.756
15	N	8537.4	0.648	< 0.002	< 0.013	2.461
18	N	14938.2	0.627	< 0.002	< 0.013	2.420
20	N	8632.3	0.606	< 0.002	< 0.013	2.671
21	N	11494.7	0.602	< 0.002	< 0.013	2.208
22	N	3759.1	0.601	< 0.002	< 0.013	3.595
23	N	6276.9	0.559	< 0.002	< 0.013	1.835
24	N	5727.4	0.558	< 0.002	< 0.013	2.573
26	N	11705.2	0.535	< 0.002	< 0.013	3.618
27	N	12471.6	0.535	< 0.002	< 0.013	3.243
28	N	5568.9	0.508	< 0.002	< 0.013	1.760
30	N	3908.0	0.502	< 0.002	< 0.013	1.464
33	N	3290.9	0.491	< 0.002	< 0.013	4.769
34	N	5552.5	0.488	< 0.002	< 0.013	1.832
35	N	5476.1	0.480	< 0.002	0.021	2.167
37	N	6221.6	0.466	< 0.002	0.021	1.665
38	N	9055.4	0.461	< 0.002	< 0.013	2.771
39	N	1889.8	0.451	< 0.002	< 0.013	2.434
40	N	3692.4	0.443	0.006	0.029	1.447
41	N	2374.2	0.441	< 0.002	0.0123	1.485
42	N	8845.7	0.439	0.004	0.021	1.520
43	N	3986.7	0.438	< 0.002	< 0.013	1.959
45	N	4066.3	0.428	< 0.002	< 0.013	1.668
46	N	6317.2	0.420	0.004	0.0201	1.417
47	N	9346.0	0.418	0.004	0.021	1.810
48	N	3221.2	0.411	< 0.002	< 0.013	1.826
49	N	3403.6	0.405	0.004	0.021	2.221
50	N	2959.8	0.400	0.008	0.033	1.819
51	N	3053.5	0.398	0.004	0.021	2.151
52	N	1835.7	0.384	< 0.002	0.013	1.419
53	N	4346.7	0.382	0.008	0.033	1.309
54	N	3213.8	0.382	0.01	0.036	1.877
56	N	4509.8	0.369	0.008	0.033	1.386
57	N	3142.6	0.368	0.006	0.029	1.995

60	N	3815.5	0.363	0.01	0.036	1.512
63	N	4254.9	0.356	0.004	0.021	1.399
64	N	8329.5	0.351	0.01	0.036	1.568
65	N	2356.4	0.348	0.008	0.033	1.546
67	N	2463.8	0.345	0.008	0.033	1.563
68	N	3514.3	0.342	0.008	0.033	1.922
70	N	2591.7	0.339	0.01	0.036	1.351
75	N	2328.0	0.333	0.01	0.036	1.590
77	N	2939.9	0.331	0.01	0.036	1.488
78	N	5257.3	0.330	0.008	0.033	1.354
84	N	11046.4	0.312	0.008	0.033	2.245
86	N	7035.6	0.307	0.01	0.036	1.809
94	N	10592.5	0.283	0.008	0.033	2.101
2	T	3437.3	-1.109	< 0.002	< 0.013	8.318
3	T	3366.4	-1.092	< 0.002	< 0.013	16.139
9	T	3481.5	-0.720	< 0.002	< 0.013	4.474
16	T	13132.9	-0.644	< 0.002	< 0.013	15.434
17	T	7549.7	-0.637	< 0.002	< 0.013	1.526
19	T	10817.5	-0.612	< 0.002	< 0.013	3.390
25	T	4211.2	-0.540	< 0.002	< 0.013	2.158
29	T	4129.1	-0.508	< 0.002	< 0.013	1.957
31	T	3321.9	-0.498	< 0.002	< 0.013	1.655
32	T	54076	-0.496	< 0.002	< 0.013	2.050
36	T	5979.6	-0.472	0.004	0.021	1.895
44	T	12670.2	-0.437	0.004	0.021	3.680
55	T	6648.8	-0.370	0.008	0.033	1.656
58	T	6565.2	-0.367	0.006	0.029	1.703
59	T	5525.2	-0.365	0.01	0.036	1.716
61	T	9226.1	-0.362	0.006	0.029	1.573

[1]The feature-specific *P*-value (Bonferroni corrected); [2]An estimate of the false discovery rate (FDR) using the procedure of Benjamini and Hochberg [30]; [3]Ratio of the mean relative ion intensity values of tumour versus normal
