# Supplementary material for: Mass spectrometry protein expression profiles in colorectal cancer tissue associated with clinico-pathological features of disease
Source: BMC Cancer. 2010 Aug 6;10:410. doi: 10.1186/1471-2407-10-410 (PMC2927547; doi:10.1186/1471-2407-10-410)
Supplement: Additional file 3 — Performance of predictive model for discriminating tumour and normal mucosa. Summary of results of optimised k-NN algorithm on an independent test dataset. [file 1471-2407-10-410-S3.PDF]

Additional file 3: Performance of predictive model for discriminating tumour and normal mucosa. The complete set of spectra from tumour and normal mucosa were randomly allocated to separate train and test datasets using default fraction allocation parameters of the SplitDataSetsTrainTest GenePattern module [27, 29]. The training set was used to optimise a model using the kNN algorithm [29] with 10 features selected using the SNR test statistic and the number of votes of the *k* neighbours weighted by cosine distance. The model was then used to predict the classification of the independent test dataset. The confidence represents the proportion of votes for the predicted class. The sensitivity and specificity of the prediction were both 1.0 (95% confidence interval: 0.679-0.992).

**Model**	**Num Data**	**Num Right**	**Num Wrong**	**Threshold**	**Num Abstain**	**[1]Abs Error**	**[2]ROC Error**
KNN	22	22	0	0	0	0	0
**[3]Specimen**	**True Class**	**Predicted Class**	**Confidence**	**Error?**			
2018T	T	T	1				
023T	T	T	1				
031T	T	T	1				
009T	T	T	1				
028T	T	T	1				
033T	T	T	1				
032T	T	T	1				
002T	T	T	1				
2044T	T	T	1				
005T	T	T	1				
003T	T	T	1				
2012NM	N	N	1				
038NM	N	N	1				
032NM	N	N	1				
017NM	N	N	1				
007NM	N	N	1				
039NM	N	N	1				
2080NM	N	N	1				
035NM	N	N	1				
020NM	N	N	1				
011NM	N	N	0.673				
028NM	N	N	0.6614				

[1]Absolute error rate; [2]Reciever operator characteristics error rate; [3]N = normal mucosa, T = tumour tissue
