# Supplementary material for: Mass spectrometry protein expression profiles in colorectal cancer tissue associated with clinico-pathological features of disease
Source: BMC Cancer. 2010 Aug 6;10:410. doi: 10.1186/1471-2407-10-410 (PMC2927547; doi:10.1186/1471-2407-10-410)
Supplement: Additional file 4 — Performance of model for predicting poor differentiation based on tumour spectra. Summary of results of 'leave-one-out' cross-validation k-NN algorithm. [file 1471-2407-10-410-S4.PDF]

Additional file 4: Performance of model for predicting poor differentiation based on tumour spectra. The KNN algorithm [29] was used in 'leave-one-out' cross-validation prediction with 2 features selected using a t-test statistic and the number of votes of the *k* neighbours weighted by cosine distance. The confidence represents the proportion of votes for the predicted class.

**Model**	**Num Data**	**Num Right**	**Num Wrong**	**Threshold**	**Num Abstain**	**[1]Abs Error**	**[2]ROC Error**
KNN	31	26	5	0	0	0.161	0.171
**Specimen**	**[3]True Class**	**Predicted Class**	**Confidence**	**Error?**			
016T	W	W	1				
021T	W	W	1				
029T	W	W	1				
034T	W	W	1				
036T	W	W	1				
2022T	W	W	1				
002T	W	W	1				
004T	P	W	1	*			
011T	P	W	1	*			
023T	P	W	1	*			
033T	W	W	0.6682				
032T	W	W	0.6678				
024T	W	W	0.667				
037T	W	W	0.6665				
2085T	W	W	0.6651				
2018T	W	W	0.6646				
017T	W	W	0.6632				
039T	W	W	0.5618				
2044T	W	W	0.5618				
028T	W	P	1	*			
026T	W	P	1	*			
005T	P	P	1				
008T	P	P	1				
031T	P	P	1				
020T	P	P	0.6718				
025T	P	P	0.6718				
2084T	P	P	0.6683				
2012T	P	P	0.6679				
009T	P	P	0.6665				
038T	P	P	0.6624				
003T	P	P	0.6617				

[1]Absolute error rate; [2]Reciever operator characteristics error rate; [3]W = well- and moderately-differentiated; P = poorly differentiated
