# Supplementary material for: Mass spectrometry protein expression profiles in colorectal cancer tissue associated with clinico-pathological features of disease
Source: BMC Cancer. 2010 Aug 6;10:410. doi: 10.1186/1471-2407-10-410 (PMC2927547; doi:10.1186/1471-2407-10-410)
Supplement: Additional file 5 — Performance of model for predicting disease recurrence based on tumour spectra. Summary of results of 'leave-one-out' cross-validation k-NN algorithm. [file 1471-2407-10-410-S5.PDF]

Additional file 5: Performance of model for predicting disease recurrence based on tumour spectra. The *k*NN algorithm [29] was used in 'leave-one-out' cross-validation prediction with 10 features selected using a t-test statistic and the number of votes of the *k* neighbours weighted by cosine distance. The confidence represents the proportion of votes for the predicted class

**Model**	**Num Data**	**Num Right**	**Num Wrong**	**Threshold**	**Num Abstain**	**[1]Abs Error**	**[2]ROC Error**
KNN	29	27	2	0	0	0.069	0.105
**Specimen**	**[3]True Class**	**Predicted Class**	**Confidence**	**Error?**			
008T	P	P	1				
024T	P	P	0.6915				
004T	P	P	0.6846				
025T	P	P	0.6652				
2012T	G	P	0.6542	*			
026T	P	P	0				
003T	G	G	1				
009T	G	G	1				
011T	G	G	1				
016T	G	G	1				
017T	G	G	1				
020T	G	G	1				
021T	G	G	1				
023T	G	G	1				
029T	G	G	1				
031T	G	G	1				
032T	G	G	1				
034T	G	G	1				
037T	G	G	1				
038T	G	G	1				
039T	G	G	1				
2022T	G	G	1				
2044T	G	G	1				
002T	P	G	1	*			
005T	G	G	0.7088				
028T	G	G	0.6738				
2084T	G	G	0.6676				
036T	G	G	0.6666				
033T	G	G	0.6429				

[1]Absolute error rate; [2]Reciever operator characteristics error rate; [3]P = poor outcome (disease recurrence); G = good outcome (disease-free)
