# Supplementary material for: Mass spectrometry protein expression profiles in colorectal cancer tissue associated with clinico-pathological features of disease
Source: BMC Cancer. 2010 Aug 6;10:410. doi: 10.1186/1471-2407-10-410 (PMC2927547; doi:10.1186/1471-2407-10-410)
Supplement: Additional file 6 — Performance of model for predicting lymph node involvement based on mucosa spectra. Summary of results of 'leave-one-out' cross-validation k-NN algorithm. [file 1471-2407-10-410-S6.PDF]

Additional file 6: Performance of model for predicting lymph node involvement based on mucosa spectra. The *k*NN algorithm [29] was used in ‘leave-one-out’ cross-validation prediction with 3 features selected using a t-test statistic and the number of votes of the *k* neighbours weighted by cosine distance. The confidence represents the proportion of votes for the predicted class.

**Model**	**Num Data**	**Num Right**	**Num Wrong**	**Threshold**	**Num Abstain**	**[1]Abs Error**	**[2]ROC Error**
KNN	33	26	7	0	0	0.212	0.212
**Specimen**	**[3]True Class**	**Predicted Class**	**Confidence**	**Error?**			
005NM	LN	LP	1	*			
037NM	LN	LP	1	*			
011NM	LP	LP	1				
016NM	LP	LP	1				
034NM	LP	LP	1				
003NM	LP	LP	1				
007NM	LP	LP	1				
039NM	LN	LP	0.696	*			
009NM	LP	LP	0.6848				
017NM	LN	LP	0.6834	*			
2085NM	LP	LP	0.6648				
2012NM	LP	LP	0.6561				
024NM	LP	LP	0.6542				
031NM	LP	LP	0.6527				
038NM	LP	LP	0.6176				
001NM	LN	LN	1				
002NM	LN	LN	1				
008NM	LN	LN	1				
023NM	LN	LN	1				
026NM	LN	LN	1				
032NM	LN	LN	1				
035NM	LN	LN	1				
2044NM	LN	LN	1				
004NM	LP	LN	1	*			
021NM	LP	LN	1	*			
028NM	LN	LN	0.6874				
020NM	LN	LN	0.6743				
2080NM	LN	LN	0.6734				
006NM	LN	LN	0.6644				
2018NM	LN	LN	0.6587				
029NM	LN	LN	0.6414				
2022NM	LN	LN	0.6414				
033NM	LP	LN	0.6207	*			

[1]Absolute error rate; [2]Reciever operator characteristics error rate; [3]LN = lymph node negative; LP = lymph node positive
